# Association of ulinastatin with 28-day mortality across different severities of viral pneumonia: a multicenter propensity score-matched study

**DOI:** 10.3389/fphar.2026.1831113

**Published:** 2026-06-30

**Authors:** Yu Zhang, Zongmian Zhang, Yunhai Zhang, Fei Tao, Baoshan Huo, Yun Wen, Wei Li, Xuezhi Lei, Jinping Zhou, Jiefu Lu, Jun Cao, Yanfei Cai, ZhuoZheng Liang, Ming Wang, Jianxiong Luo, Songtao Liu, Li Zhang, Peng Zheng, Mengjia Liu, Lixin Zhou, Xinhua Qiang

**Affiliations:** 1 Department of Critical Care Medicine, The First People’s Hospital of Foshan (The Affiliated Foshan Hospital of Southern University of Science and Technology), School of Medicine, Southern University of Science and Technology, Foshan, Guangdong, China; 2 The Sixth Affiliated Hospital, South China University of Technology, Foshan, Guangdong, China; 3 Department of Critical Care Medicine, Foshan Hospital of Traditional Chinese Medicine (The Eighth Clinical Medical College of Guangzhou University of Chinese Medicine), Foshan, Guangdong, China; 4 Foshan Fosun Chancheng Hospital, Foshan, Guangdong, China; 5 The Second People’s Hospital of Foshan, Foshan, Guangdong, China

**Keywords:** COVID-19, critical illness, influenza, mortality, observational study, ulinastatin

## Abstract

**Introduction:**

Hyperinflammation drives mortality in severe or critical viral pneumonia. We evaluated the association of ulinastatin, an immunomodulatory agent, with 28 day mortality in patients with severe or critical pneumonia caused by COVID-19 or influenza.

**Methods:**

In this multicenter, retrospective cohort study, we included adult patients admitted to the intensive care unit (ICU) with confirmed SARS-CoV-2 or influenza infection. To control for confounding, 1:1 propensity score matching (PSM) was performed.

**Results:**

Of 330 eligible patients, 194 were included in the final matched cohort. In the overall matched cohort, ulinastatin treatment did not significantly reduce 28 day all-cause mortality compared with the control group (29.9% vs. 38.1%; HR 0.69, 95% CI 0.43 to 1.12, p = 0.128). However, exploratory subgroup analysis revealed that the survival benefit was driven predominantly by the critically ill subgroup (HR 0.51, 95% CI 0.30 to 0.87, p = 0.011), with no benefit observed in severe patients (HR 1.48, 95% CI 0.45 to 4.85, p = 0.513). This protective association in critical patients was consistent across COVID-19 and influenza etiologies (P for interaction = 0.313) and was not significantly modified by baseline metabolic comorbidities (all P for interaction > 0.05).

**Conclusion:**

Ulinastatin treatment did not significantly improve 28 day survival in the overall cohort of severe-to-critical viral pneumonia. However, exploratory subgroup analyses suggest a potential survival benefit specifically in critically ill patients, predominantly driven by the COVID-19 subset. Further studies are needed to confirm these findings across specific viral etiologies. These hypothesis-generating findings highlight the need for precision immunomodulation targeting the optimal therapeutic window.

## 1 Introduction

Severe or critical viral pneumonia is mainly caused by pathogens, such as SARS-CoV-2 and influenza viruses, and is one of the major causes of mortality in intensive care units (ICU) worldwide. Although the viral pathogens are different, the common pathological feature of these severe infections is the dysregulation of the host immune response, which usually ends up as a cytokine storm. This state of hyperinflammation, rather than the direct cytotoxicity of the virus, is considered a key driver of disease progression to acute respiratory distress syndrome (ARDS), multiple organ dysfunction, and death (Pelaia et al., 2020; Polidoro et al., 2020; Ragab et al., 2020; Wang et al., 2021). Therefore, developing host-directed immunomodulatory therapies effective against diverse viral threats remains a crucial but unresolved challenge for critical care and infectious disease research.

The coexistence of excessive inflammatory pathways in severe COVID-19 and influenza provides a strong theoretical basis for exploring immunomodulatory agent. Ulinastatin is a serine protease inhibitor with potent anti-inflammatory properties, and it is one such candidate drug (Muramatu et al., 1980). Ulinastatin is known to reduce key inflammatory biomarkers, including C-reactive protein (CRP), interleukin-6 (IL-6), and tumor necrosis factor-α (TNF-α). It has shown potential in the treatment of diseases such as sepsis and ARDS, which share similar pathophysiological features with severe or critical viral pneumonia (Liu et al., 2017; Zhang et al., 2019; Meng et al., 2020). Interestingly, more than a decade ago, the potential use of ulinastatin as a host-directed therapy for severe influenza was proposed. This was based on the fact that ulinastatin can protect the integrity of lysosomes and inhibit the damaging proteases released during influenza virus infection (Yuan, 2013). Although the mechanism of action of ulinastatin has been known for a long time, and similar hypotheses exist in the treatment of COVID-19 (Mehta et al., 2020), the evidence for its clinical application remains scattered and controversial. While some smaller observational studies suggest that ulinastatin may have potential benefits (Huang et al., 2022), other retrospective analyses, particularly in the context of COVID-19, have not found a significant effect on mortality (Jain et al., 2022; Mehta et al., 2023).

These conflicting findings raise a crucial question: is the therapeutic association of ulinastatin with outcomes in severe or critical viral pneumonia universal, or is it dependent on specific viruses or patient clinical phenotypes? To resolve this uncertainty, we need direct comparative evidence. Therefore, this study aimed to fill this research gap by assessing the association between ulinastatin and 28 day mortality in patients with severe or critical pneumonia caused by COVID-19 or influenza. The study subjects were a large real-world, multicenter cohort. Our aim is specifically to explore the heterogeneity of this association across different viral pathogens and baseline disease severities. This will provide a basis for developing more precise, time-sensitive immunomodulatory strategies for this patient population in the future.

## 2 Materials and methods

### 2.1 Study design and participants

This was a multicenter, retrospective cohort study conducted at five tertiary hospitals in Foshan, China, included the First People’s Hospital of Foshan, the Sixth Affiliated Hospital, South China University of Technology, Foshan Hospital of Traditional Chinese Medicine, Foshan Fosun Chancheng Hospital and the Second People’s Hospital of Foshan. Clinical records from all consecutive adult subjects admitted to ICU with a diagnosis of severe or critical pneumonia caused by either confirmed SARS-CoV-2 or influenza (type A or B) virus infection from January 2022 to June 2024 were retrospectively reviewed. Data were manually extracted from the electronic medical record system. As this study was a retrospective analysis using secondary data without any personal information, the requirement for obtaining informed consent was waived and the study design was approved by the First People’s Hospital of Foshan clinical research ethics committee (approval number: FSYYY 2023-163).

According to historical medical records, all patients confirmed with severe or critical pneumonia caused by either confirmed SARS-CoV-2 or influenza (type A or B) virus infection who received ulinastatin were assigned to the ulinastatin cohort. Key treatment parameters, including the initiation time, single-dose amount, and treatment duration, were systematically recorded. Patients who did not receive ulinastatin were assigned to the conventional cohort. According to their conditions, patients received appropriate standard care measures including systemic steroid, supplemental oxygen, invasive or non-invasive mechanical ventilation and other organ function support, at the discretion of the responsible clinical team.

Because of the real-world, retrospective nature of this study, the initiation, dosage, and duration of ulinastatin therapy were not strictly protocolized but were clinician-dependent. Treatment decisions were guided by the attending physicians based on the severity of the inflammatory response and the patients’ overall clinical trajectory. The standard dosing regimen typically ranged from 100,000 to 200,000 units administered intravenously two to three times daily.

### 2.2 Definitions

#### 2.2.1 Virus infection confirmation

A definitive diagnosis of COVID-19 required patients to exhibit typical clinical symptoms alongside laboratory-confirmed SARS-CoV-2 infection. Virological evidence included any of the following: detection of viral RNA via RT-PCR from respiratory tract swabs (nasopharyngeal or oropharyngeal), a positive rapid antigen assay, or successful viral culture. COVID-19 is divided into four clinical classifications as following mild, moderate, severe and critical according to its severity (Released by National Health Commission of People’s Republic of China & National Administration of Traditional Chinese Medicine on 5 January 2023). Influenza infection was confirmed by the detection of influenza A or B virus via reverse transcription-polymerase chain reaction (RT-PCR), or a positive rapid antigen test on a nasopharyngeal swab specimen, accompanied by clinical manifestations of an influenza-like illness.

#### 2.2.2 Severity classification of COVID-19

Patients were classified as having severe COVID-19 if they exhibited marked tachypnea (respiratory rate ≥30 breaths per minute), severe hypoxemia (resting oxygen saturation ≤ 93% or P_a_O_2_/ F_i_O_2_ ratio ≤ 300 mmHg), or rapid radiographic deterioration (> 50% lesion expansion within 24–48 h), cannot be explained by a cause other than SARS-CoV-2 infection (Released by National Health Commission of People’s Republic of China & National Administration of Traditional Chinese Medicine on 5 January 2023). The critical COVID-19 designation was reserved for individuals presenting with life-threatening complications, such as hemodynamic instability (shock), the necessity for mechanical ventilation due to respiratory failure, or the onset of extra-pulmonary organ dysfunction mandating intensive care (Released by National Health Commission of People’s Republic of China & National Administration of Traditional Chinese Medicine on 5 January 2023).

#### 2.2.3 Severity classification of influenza

Severe influenza was defined as any criteria of the following: (1) shortness of breath or difficulty breathing; (2) resting oxygen saturation ≤93% on ambient air; (3) P_a_O_2_/F_i_O_2_ ≤300 mmHg; (3) chest imaging showing multilobar infiltrates or progression of lesions >50% within 24–48 h; (4) evidence of significant exacerbation of underlying chronic medical conditions (Author Anonymous, 2010; Kim et al., 2011; Uyeki et al., 2019). Critical influenza was defined as any criteria of the following: (1) respiratory failure requiring mechanical ventilation, including ARDS; (2) shock with the need for vasopressor support; (3) other organ failure requiring ICU monitoring (Author Anonymous, 2010; Kim et al., 2011; Uyeki et al., 2019).

Although there are subtle differences between the specific criteria, both pathogens define critical illness as a clinically equivalent state of organ dysfunction, such as ARDS, shock, or the need for mechanical ventilation.

### 2.3 Inclusion and exclusion criteria

The inclusion criteria were that all adult patients were admitted to the hospital with confirmed severe or critical COVID-19 or influenza. The exclusion criteria were as follows: (1) concomitant malignancy, autoimmune disease; (2) immunosuppression; (3) patient died within 48 h of hospitalization; (4) patient whose clinical data were incomplete; (5) patients who anticipated to be discharged, transferred to another hospital; (6) patients who received ulinastatin less than 2 days. To prevent immortal time bias, early deaths (<48 h) were strictly excluded from all groups prior to matching, inherently establishing a 48 h landmark cohort for subsequent survival analyses.

### 2.4 Data collection and outcome

Clinical information for all patients was extracted from the hospital’s electronic medical record system. The following variables were recorded for each patient: age, gender, BMI, comorbidities, APACHE II score, SOFA score, laboratory, treatment administered, mortality and hospital-free days. The primary outcome was all-cause mortality at 28 days. Secondary outcomes included length of hospital stay, length of ICU stay, the rate of extra-corporeal membrane oxygenation (ECMO), the rate of mechanical ventilation, or the rate of continuous renal replacement therapy (CRRT). For the purpose of survival evaluation, ulinastatin was analyzed as a time-fixed exposure in all models.

The 28 day survival status was initially tracked via the electronic medical record system. For patients discharged alive prior to day 28, outcome ascertainment was completed through structured telephone interviews with the patients or their immediate family members. Patients who were lost to follow-up during the 28 day period were right-censored at the date of their last confirmed clinical contact or successful communication for all survival analyses.

Regarding the handling of missing data, specific laboratory variables were missing primarily because tests were ordered based on routine clinical indications rather than a standardized protocol. To prevent missing data from introducing bias into the matching process, individual laboratory variables with substantial missingness were excluded from the propensity score model. Instead, disease severity was balanced using the APACHE II and SOFA scores, which were complete for all analyzed patients. For descriptive statistics, an available-case analysis approach was utilized, analyzing data based on the non-missing observations without applying imputation.

### 2.5 Statistical analysis

Statistical analyses were performed using SPSS version 26.0 and R 4.3.3. A two-sided p-value <0.05 was considered statistically significant. To mitigate confounding variables and baseline covariate discrepancies, a 1:1 propensity score matching (PSM) was performed. The propensity score, representing the probability of receiving ulinastatin treatment, was calculated for each patient using a multivariable logistic regression model. The variables included in the model were: age, gender, BMI, virus type (COVID-19 vs. influenza), baseline APACHE II score, baseline SOFA score, and the presence of the following major comorbidities: hypertension, diabetes, coronary heart disease, chronic respiratory diseases, chronic kidney diseases, chronic liver disease, cerebrovascular disease. Patients in the ulinastatin group were matched to patients in the control group using a 1:1 nearest-neighbor matching algorithm with a caliper width of 0.2 of the standard deviation of the logit of the propensity score. The balance of baseline covariates before and after matching was assessed using standardized mean differences (SMDs), with an SMD < 0.20 considered as an indicator of adequate baseline balance. In the matched cohort, continuous variables were compared using the Mann-Whitney U test, and categorical variables were compared using Pearson’s Chi-squared test or Fisher’s exact test. The primary outcome was analyzed using the Kaplan-Meier method with the log-rank test. Unadjusted and multivariable Cox proportional hazards models were used to estimate hazard ratios (HRs) and 95% confidence intervals (CIs). The proportional hazards assumption, verified via Schoenfeld residuals and log-minus-log plots, was fully satisfied (all p > 0.05). To address residual confounding, a doubly robust estimation (multivariable Cox regression) adjusted for timing to ICU admission, vasopressor requirement, ARDS severity, and bacterial co-infection was conducted as a sensitivity analysis in the critically ill cohort. Subgroup analyses and tests for interaction were also performed within the matched cohort. All subgroups were prespecified by the research team prior to data analysis. Due to their exploratory nature, no multiplicity adjustments were applied, rendering these findings hypothesis-generating.

## 3 Results

### 3.1 Study population and baseline characteristics

**FIGURE 1:**
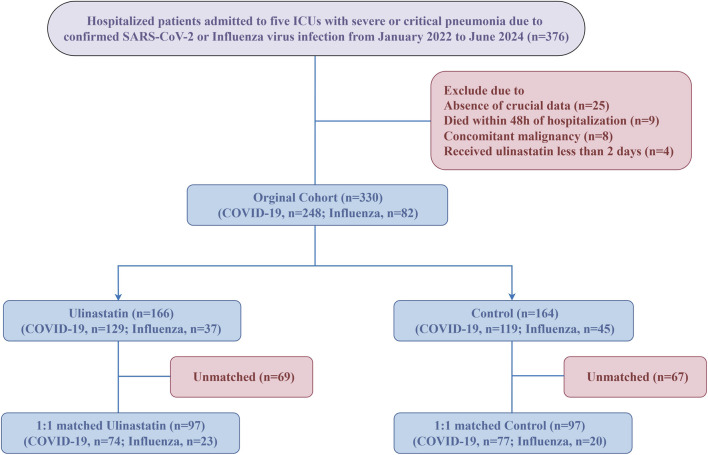
The cohort flow diagram.

A total of 376 patients with severe or critical viral pneumonia were screened. After excluding 46 patients based on pre-specified criteria, 330 patients were included in the primary cohort, comprising 248 (75.2%) with COVID-19 and 82 (24.8%) with influenza (Figure 1). Within this cohort, 166 patients received ulinastatin and 164 were in the control group. To further enhance comparability and minimize potential selection bias, a 1:1 PSM was performed. A final matched cohort of 194 patients (97 in each group) was included in the primary analysis. In the matched cohort, intensive follow-up was conducted for 28 day outcomes. Among the patients discharged alive within 28 days, 17 were lost to follow-up due to refusal or inability to be contacted. For the remaining 75 successfully followed discharged patients, five out-of-hospital deaths were recorded within the 28 day window, ensuring a robust representation of the primary endpoint.

The baseline demographic and clinical characteristics of the patients before and after matching are presented in Table 1. Before matching, patients in the ulinastatin and control groups were generally well-balanced. After PSM, all baseline covariates, including age, gender, BMI, virus type, APACHE II score, SOFA score, concomitant systemic steroid, comorbidities and key laboratory parameters were well-balanced between the two groups, and all standardized mean differences (SMDs) being < 0.20 (Supplementary Table S1).

**TABLE 1:** Baseline characteristics of the study population. Values are Median [IQR] unless stated otherwise.

Baseline characteristics	Before matching				After propensity score matching (1:1)			
	Overall, N = 330	Control, N = 164	Ulinastatin, N = 166	p-value	Overall, N = 194	Control, N = 97	Ulinastatin, N = 97	*p*-value
Age, years	73.5 [64.0, 81.0]	72.0 [62.5, 81.0]	74.0 [65.0, 82.0]	0.308	73.0 [64.0, 82.0]	72.0 [61.0, 82.0]	74.0 [66.0, 82.0]	0.330
Gender, n (%)	​	​	​	0.686	​	​	​	0.349
Male	229 (69.4%)	116 (70.7%)	113 (68.1%)	​	135 (69.6%)	64 (66.0%)	71 (73.2%)	​
Female	101 (30.6%)	48 (29.3%)	53 (31.9%)	​	59 (30.4%)	33 (34.0%)	26 (26.8%)	​
BMI, kg/m2, mean ± SD	24.5 ± 3.2	24.5 ± 3.3	24.6 ± 3.1	0.811	24.4 ± 3.1	24.4 ± 3.3	24.5 ± 3.0	0.872
Virus type, n (%)	​	​	​	0.340	​	​	​	0.730
COVID-19	248 (75.2%)	119 (72.6%)	129 (77.7%)	​	151 (77.8%)	77 (79.4%)	74 (76.3%)	​
Influenza	82 (24.8%)	45 (27.4%)	37 (22.3%)	​	43 (22.2%)	20 (20.6%)	23 (23.7%)	​
APACHE II score	21.0 [15.0, 27.0]	21.0 [15.0, 27.0]	21.5 [16.0, 27.0]	0.746	21.0 [15.0, 27.0]	21.0 [14.0, 26.0]	21.0 [15.0, 27.0]	0.824
SOFA score	6.0 [4.0, 9.0]	6.0 [4.0, 9.0]	7.0 [5.0, 9.0]	0.054	6.0 [4.0, 8.3]	6.0 [4.0, 9.0]	6.0 [5.0, 8.0]	0.964
Time from diagnosis to treatment, days	0.0 [0.0, 2.0]	NA	0.0 [0.0, 2.0]	NA	0.0 [0.0, 2.0]	NA	0.0 [0.0, 2.0]	NA
Daily dose of ulinastatin, 10^4^U	30 [30, 60]	NA	30 [30, 60]	NA	30 [30, 60]	NA	30 [30, 60]	NA
Duration of treatment, days	7 [5, 14]	NA	7 [5, 14]	NA	9 [5, 14]	NA	9 [5, 14]	NA
Concomitant systemic steroid, n (%)	181 (54.8%)	86 (52.4%)	95 (57.2%)	0.445	94 (48.5%)	46 (47.4%)	48 (49.5%)	0.886
Comorbidities, n (%)	​	​	​	​	​	​	​	​
Hypertension	128 (38.8%)	61 (37.2%)	67 (40.4%)	0.633	76 (39.2%)	35 (36.1%)	41 (42.3%)	0.462
Diabetes	84 (25.5%)	41 (25.0%)	43 (25.9%)	0.951	47 (24.2%)	24 (24.7%)	23 (23.7%)	>0.999
Coronary heart disease	60 (18.2%)	31 (18.9%)	29 (17.5%)	0.846	36 (18.6%)	19 (19.6%)	17 (17.5%)	0.853
Chronic respiratory diseases	37 (11.2%)	19 (11.6%)	18 (10.8%)	0.969	21 (10.8%)	11 (11.3%)	10 (10.3%)	>0.999
Chronic kidney diseases	47 (14.2%)	27 (16.5%)	20 (12.1%)	0.322	26 (13.4%)	11 (11.3%)	15 (15.5%)	0.527
Chronic liver disease	10 (3.0%)	6 (3.7%)	4 (2.4%)	0.541	5 (2.6%)	2 (2.1%)	3 (3.1%)	>0.999
Cerebrovascular disease	70 (21.2%)	36 (22.0%)	34 (20.5%)	0.848	38 (19.6%)	16 (16.5%)	22 (22.7%)	0.366
Laboratory results	​	​	​	​	​	​	​	​
WBC, 10^9^/L (n = 162/164‡, n = 97/96§)	10.5 [7.8, 14.6]	10.6 [8.1, 13.9]	10.3 [7.2, 15.1]	0.779	10.6 [8.0, 15.3]	10.6 [8.3, 13.6]	10.9 [7.9, 16.5]	0.661
Neutrophils, 10^9^/L (n = 162/164‡, n = 97/96§)	9.1 [6.3, 12.8]	9.2 [6.8, 12.5]	8.9 [6.1, 13.0]	0.664	9.4 [7.0, 12.7]	9.6 [7.0, 12.1]	7.1 [9.1, 13.0]	0.897
Lymphocytes, 10^9^/L (n = 162/164‡, n = 97/96§)	0.60 [0.36, 0.96]	0.59 [0.37, 0.94]	0.61 [0.36, 0.96]	0.796	0.59 [0.37, 0.96]	0.58 [0.37, 0.90]	0.69 [0.38, 1.02]	0.262
Hemoglobin, g/L (n = 162/164‡, n = 97/96§)	107 [85, 127]	107 [86, 125]	109 [85, 130]	0.559	107 [83, 129]	104 [79, 125]	111 [88, 132]	0.184
Platelets, 10^9^/L (n = 162/164‡, n = 97/96§)	191 [125, 255]	201 [137, 275]	187 [121, 245]	0.242	193 [123, 252]	201 [141, 261]	189 [120, 244]	0.317
CRP, mg/L (n = 121/126‡, n = 77/73§)	118 [55, 158]	116 [56, 146]	122 [51, 176]	0.500	117 [56, 154]	113 [58, 147]	118 [45, 161]	0.855
PCT, ng/mL (n = 149/144‡, n = 90/82§)	1.2 [0.4, 5.9]	1.0 [0.3, 5.2]	1.4 [0.4, 7.7]	0.162	0.9 [0.3, 5.1]	0.8 [0.3, 3.3]	1.4 [0.4, 5.4]	0.097
ALT, U/L (n = 154/156‡, n = 91/93§)	25 [16, 45]	26 [17, 49]	25 [16, 43]	0.560	24 [16, 48]	24 [15, 48]	25 [17, 47]	0.575
AST, U/L (n = 141/142‡, n = 87/83§)	38 [25, 64]	40 [25, 66]	36 [26, 62]	0.527	37 [23, 63]	36 [22, 63]	38 [27, 62]	0.373
TBIL, μmol/L (n = 150/155‡, n = 89/93§)	11.6 [7.8, 18.5]	11.4 [7.5, 16.9]	12.0 [8.0, 20.3]	0.302	12.1 [7.8, 20.1]	11.6 [7.0, 17.3]	12.5 [8.5, 21.6]	0.137
Albumin, g/L (n = 142/140‡, n = 88/82§)	31.3 [27.9, 35.0]	31.3 [27.9, 35.0]	31.2 [27.9, 35.0]	0.762	31.0 [27.7, 35.8]	31.1 [27.8, 35.7]	30.8 [27.7, 35.9]	0.985
Creatinine, μmol/L (n = 156/159‡, n = 91/94§)	111 [70, 221]	118 [68, 214]	108 [72, 229]	0.777	113 [72, 229]	121 [72, 210]	108 [72, 239]	0.695

ALT, alanine aminotransferase; APACHE, acute physiology and chronic health evaluation; AST, aspartate aminotransferase; BMI, body mass index; CRP = C-reactive protein; PCT, procalcitonin; SOFA, sequential organ failure assessment; TBIL, total bilirubin; WBC, white blood cells.

‡ Number of patients in control group/number in ulinastatin group before matching.

§ Number of patients in control group/number in ulinastatin group after matching.

### 3.2 Primary outcome

**FIGURE 2:**
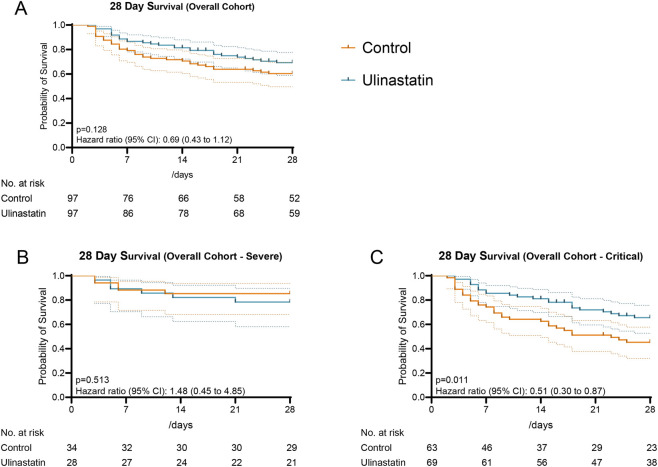
Kaplan-Meier estimates for the primary outcome of 28 day mortality in the overall matched cohort and subgroups stratified by disease severity. **(A)** Survival curves for the overall matched cohort (n = 194). **(B)** Survival curves for the subgroup of patients with severe pneumonia at admission. **(C)** Survival curves for the subgroup of patients with critical pneumonia at admission. P-values were calculated using the log-rank test. Hazard ratios (HR) and 95% confidence intervals (CI) were derived from Cox proportional hazards models.

In the primary analysis of the overall matched cohort (n = 194), although the crude 28 day mortality was numerically lower in the ulinastatin group (29.9% vs. 38.1%, *p* = 0.289), survival analysis revealed that this overall association did not reach statistical significance (HR 0.69, 95% CI 0.43 to 1.12, *p* = 0.128) (Figure 2A).

However, to explore the potential source of therapeutic response, we performed pre-specified subgroup analyses stratified by baseline disease severity. Strikingly, ulinastatin was associated with a significant reduction in the hazard of death specifically in the subgroup of critically ill patients (n = 132, HR 0.51, 95% CI 0.30 to 0.87, *p* = 0.011) (Figure 2C). This association was not observed in the severe group (n = 62, HR 1.48, 95% CI 0.45 to 4.85, *p* = 0.513) (Figure 2B). The interaction test between treatment and disease severity was not statistically significant (interaction P value = 0.104). Importantly, to mitigate the influence of residual confounding after matching, we performed a doubly robust estimation adjusting for additional critical illness parameters. Antiviral therapy was highly prevalent (94.7%) in this critical subset and was excluded from the multivariable model to avoid sparse-data bias. As shown in Supplementary Table S2, even after adjusting for timing to ICU admission, vasopressor requirement, ARDS severity, and bacterial co-infection, the survival benefit of ulinastatin in critically ill patients remained robust and statistically significant (Adjusted HR = 0.46, 95% CI 0.26 to 0.82, *p* = 0.009).

**FIGURE 3:**
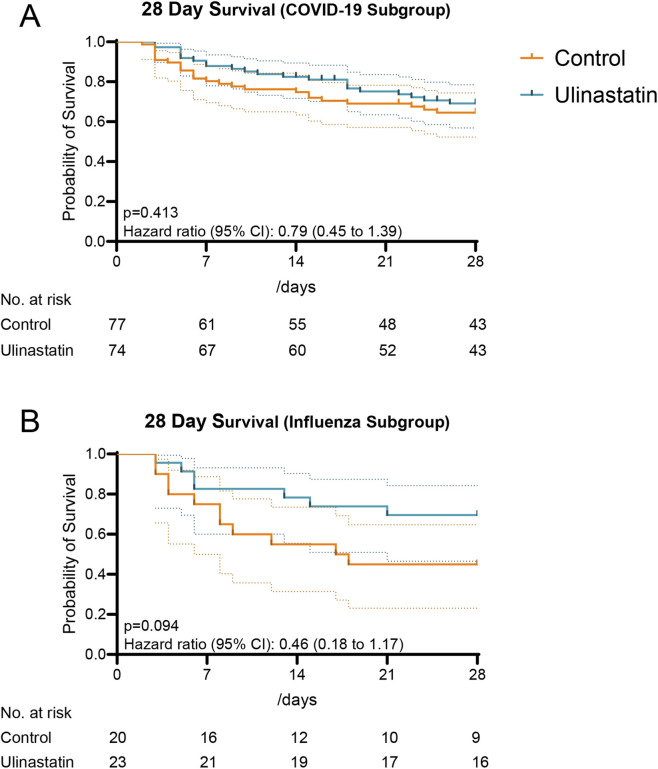
Kaplan-Meier estimates of 28 day survival stratified by viral etiology. **(A)** Survival curves for the subgroup of patients with COVID-19 (n = 151). **(B)** Survival curves for the subgroup of patients with influenza (n = 43). HR, hazard ratio; CI, confidence interval.

Next, we explored whether this association depends on the specific viral pathogen. As shown in Figure 3, consistent protective trends were observed in both the COVID-19 subgroup (n = 151, HR 0.79, 95% CI 0.45 to 1.39, *p* = 0.413) and the influenza subgroup (n = 43, HR 0.46, 95% CI 0.18 to 1.17, *p* = 0.094). The interaction test indicated no significant difference between the two viral etiologies (P for interaction = 0.313).

### 3.3 Subgroup analysis

**FIGURE 4:**
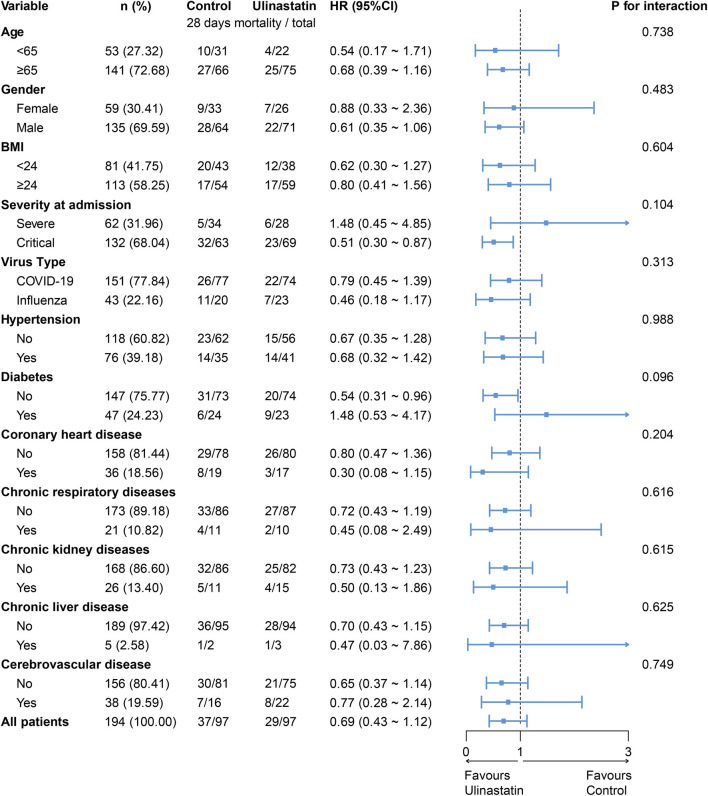
Forest plot of subgroup analyses for 28 day mortality. The plot displays the hazard ratios (HR) and 95% confidence intervals (CI) for ulinastatin versus control across pre-specified and exploratory subgroups in the matched cohort. The P value for interaction indicates the statistical significance of the difference in treatment effect between subgroups. * NE, not estimable, due to zero events in the control group.

The heterogeneity of treatment associations was further evaluated across key clinical subgroups using interaction analysis (Figure 4). After rigorous propensity score matching and complete follow-up, no statistically significant interactions were observed between ulinastatin treatment and major metabolic comorbidities, including hypertension (P for interaction = 0.988), diabetes (P for interaction = 0.096), coronary heart disease (P for interaction = 0.204), chronic respiratory diseases (P for interaction = 0.616), chronic kidney diseases (P for interaction = 0.615), chronic liver disease (P for interaction = 0.625) and cerebrovascular disease (P for interaction = 0.749). This suggests that the potential survival benefit observed in the critical phase is largely independent of patients’ baseline chronic metabolic status.

### 3.4 Secondary and other outcomes

Secondary efficacy outcomes are summarized in Table 2. There were no statistically significant differences in length of hospital stay, length of ICU stay, the rate of extra-corporeal membrane oxygenation (ECMO), the rate of mechanical ventilation, or the rate of continuous renal replacement therapy (CRRT) between the two groups. Similarly, the dynamic trajectories of key inflammatory and organ function markers over the first 7 days, showed no significant differences between the ulinastatin and control groups (Supplementary Figure S1).

**TABLE 2:** Other efficacy outcomes for the ulinastatin and control groups. Values are median [IQR] unless stated otherwise.

Outcomes	Overall, N = 194	Control, N = 97	Ulinastatin, N = 97	*p*-value
Hospital stays, days	22.0 [14.0, 32.0]	19.0 [13.0, 31.0]	24.0 [16.0, 32.0]	0.104
ICU stays, days	12.5 [7.0, 20.0]	12.0 [6.0, 19.0]	13.0 [7.0, 21.0]	0.311
ECMO, n (%)	4 (2.1%)	1 (1.0%)	3 (3.1%)	0.312
Mechanical ventilation, n (%)	170 (87.6%)	87 (89.7%)	83 (85.6%)	0.513
CRRT, n (%)	64 (33.0%)	37 (38.1%)	27 (27.8%)	0.169

CRRT, continuous renal replacement therapy; ECMO, extra-corporeal membrane oxygenation.

## 4 Discussion

In this multicenter propensity score-matched study, we evaluated the use of ulinastatin in patients with severe or critical viral pneumonia. Our primary finding is that while ulinastatin treatment did not significantly improve 28 day survival in the overall cohort, exploratory analysis identified a distinct therapeutic window: a significant survival benefit was observed exclusively in critically ill patients. Furthermore, this potential benefit appears to be independent of specific viral etiology and baseline chronic metabolic comorbidities.

The observation that the survival benefit was confined to the critical subgroup (HR 0.51), rather than the severe subgroup (HR 1.48), which supports the concept of a therapeutic window for immune modulation. Ulinastatin exerts its effects by inhibiting pro-inflammatory proteases and mitigating systemic cytokine storms (Muramatu et al., 1980; Zhang et al., 2019). In severe patients, the inflammatory response may still be largely localized or functionally compensatory. Thus, broad pharmacological inhibition might be biologically redundant or even hinder viral clearance. Conversely, in critical patients, the host response shifts to a dysregulated, hyperinflammatory state, such as septic shock or ARDS (Pu et al., 2017; Hojyo et al., 2020). It is in this phase of acute decompensation, that the braking effect of ulinastatin appears to be most clinically valuable, potentially preventing irreversible organ damage.

Notably, our interaction analysis demonstrated that the efficacy of ulinastatin was not significantly modified by the specific viral pathogen (COVID-19 vs. influenza, P for interaction = 0.313). This provides vital evidence for the concept of “pan-viral” host-directed therapies. It suggests that ulinastatin targets the shared downstream pathway in severe viral pathogenesis, the host protease-mediated inflammatory cascade, rather than the virus itself (Cobb et al., 2021; Zhou et al., 2024). However, the distinct inflammatory kinetics of these viruses must be acknowledged. Given the limited sample size of the influenza subgroup (n = 43), our study was underpowered for definitive pathogen-specific inference. Therefore, the observed benefits should primarily be attributed to the predominantly COVID-19 cohort, and the efficacy of ulinastatin in other specific viral pneumonias warrants further large-scale investigation. Furthermore, after robust matching and follow-up, major comorbidities such as hypertension and diabetes did not significantly influence treatment efficacy. This implies that acute severity, rather than chronic metabolic background, is the primary determinant of ulinastatin’s clinical utility.

Despite the survival benefit, we observed no significant differences in dynamic changes in routine laboratory markers or secondary outcomes. This dissociation may imply that ulinastatin’s mechanism of action involves pathways not captured by standard clinical tests, such as the protection of the endothelial glycocalyx, inhibition of microthrombosis, or stabilization of lysosomal membranes (Li et al., 2015). The therapeutic effect could be time-dependent, a notion supported by a previous study suggesting greater benefit with longer treatment durations (Liu et al., 2024). Additionally, the observed trend toward longer hospital stays in the ulinastatin group may reflect a survival bias, meaning that patients who would have otherwise died early survived to require prolonged recovery.

Our study is subject to several limitations, many of which are inherent to retrospective designs. First, although we employed rigorous propensity score matching and further doubly robust estimation to adjust for critical illness parameters, unmeasured variables could still influence the outcomes, such as detailed mechanical ventilation settings, dynamic changes in clinical trajectories prior to ICU admission, or subtle variations in physician decision-making. Therefore, the causal inference drawn from this study should be interpreted with appropriate caution. Second, although we utilized a strict 48-h landmark approach to effectively mitigate immortal time bias, the exact hour of ulinastatin initiation varied among patients. As a retrospective study, inherent temporal biases related to the precise timing of clinical interventions cannot be definitively eliminated. Third, the exclusion of patients who died within 48 h of hospitalization might theoretically introduce selection bias by removing the most fulminant cases. However, this criterion was necessary because such early mortality is typically driven by an irreversible disease trajectory established prior to admission, rendering any immunomodulatory intervention futile. Fourth, we lacked data on specific immunological biomarkers (e.g., IL-6, TNF-α, lymphocyte subsets) to directly verify the immunomodulatory mechanism. Finally, our subgroup findings regarding disease severity and viral etiology are exploratory and hypothesis-generating in nature. Because no formal multiplicity correction was applied for the multiple subgroup comparisons, there is an increased risk of Type I error, and the significant findings in the critical subgroup might represent a chance finding. This further emphasizes the necessity for future large-scale, prospective randomized controlled trials to validate these observations.

## 5 Conclusion

Ulinastatin did not significantly reduce 28 day mortality in the overall population of severe-to-critical viral pneumonia. The primary endpoint of this matched cohort was negative. However, exploratory analyses suggest it may provide a substantial survival benefit specifically in critically ill patients. While further adequately powered studies are needed to determine its efficacy across distinct viral etiologies, these hypothesis-generating findings highlight the necessity of precision immunomodulation targeting the hyperinflammatory phase, and warrant further validation in prospective trials focusing on this specific critical phenotype.

## Data Availability

The raw data supporting the conclusions of this article will be made available by the authors, without undue reservation.
