# Supplementary material for: Association of ulinastatin with 28-day mortality across different severities of viral pneumonia: a multicenter propensity score-matched study
Source: Front Pharmacol. 2026 Jun 30;17:1831113. doi: 10.3389/fphar.2026.1831113 (PMC13364561; doi:10.3389/fphar.2026.1831113)
Supplement: Supplementary file 1 [file Supplementaryfile1.docx]

**Supplementary Figure**

**Supplementary Figure S1.** Dynamic changes of key laboratory parameters over the first 7 days. Trajectories of hematological and biochemical markers in the ulinastatin and control groups from Day 0 to Day 7. (A) White blood cells; (B) Neutrophils; (C) Lymphocytes; (D) Hemoglobin; (E) Platelets; (F) C-reactive protein (CRP); (G) Procalcitonin (PCT); (H) Alanine aminotransferase (ALT); (I) Aspartate aminotransferase (AST); (J) Total bilirubin (TBIL); (K) Albumin; (L) Creatinine. Data are presented as mean (error bars indicate standard error).


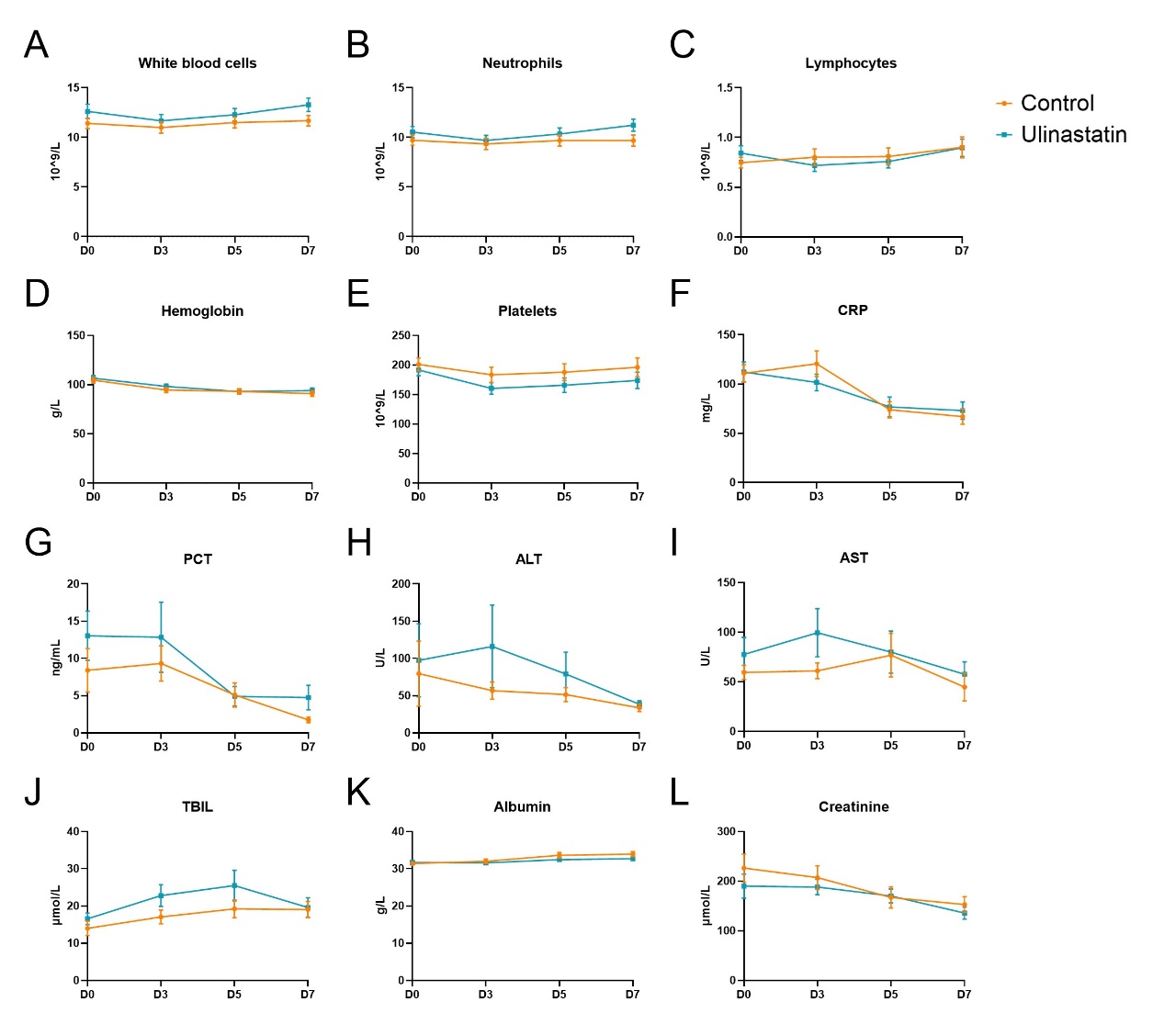


**Supplementary Table**

**Supplementary Table S1.** Baseline characteristics and Standardized Mean Differences (SMDs) before and after propensity score matching.

| Variables | Control (n=97) | Ulinastatin (n=97) | *p*-value | SMD  Before Matching | SMD  After Matching |
| --- | --- | --- | --- | --- | --- |
| Age, years (mean ± SD) | 69.76 ± 14.84 | 72.02 ± 13.45 | 0.268 | 0.112 | 0.159 |
| Gender |  |  | 0.349 | 0.058 | 0.157 |
| Female, n (%) | 33 (34.0) | 26 (26.8) |  |  |  |
| Male, n (%) | 64 (66.0) | 71 (73.2) |  |  |  |
| BMI (mean ± SD) | 24.39 ± 3.29 | 24.46 ± 3.01 | 0.872 | 0.026 | 0.023 |
| Virus type, n (%) |  |  | 0.73 | 0.119 | 0.075 |
| COVID-19 | 77 (79.4) | 74 (76.3) |  |  |  |
| Influenza | 20 (20.6) | 23 (23.7) |  |  |  |
| APACHE II score (mean ± SD) | 21.15 ± 8.32 | 21.20 ± 7.42 | 0.971 | 0.011 | 0.005 |
| SOFA score (mean ± SD) | 6.73 ± 3.20 | 6.86 ± 3.34 | 0.792 | 0.196 | 0.038 |
| Comorbidities, n (%) |  |  |  |  |  |
| Hypertension | 35 (36.1) | 41 (42.3) | 0.462 | 0.065 | 0.127 |
| Diabetes mellitus | 24 (24.7) | 23 (23.7) | 1 | 0.021 | 0.024 |
| Coronary heart disease | 19 (19.6) | 17 (17.5) | 0.853 | 0.037 | 0.053 |
| Chronic respiratory disease | 11 (11.3) | 10 (10.3) | 1 | 0.024 | 0.033 |
| Chronic kidney disease | 11 (11.3) | 15 (15.5) | 0.527 | 0.127 | 0.121 |
| Chronic liver disease | 2 (2.1) | 3 (3.1) | 1 | 0.073 | 0.065 |
| Cerebrovascular disease | 16 (16.5) | 22 (22.7) | 0.366 | 0.036 | 0.156 |

**Supplementary Table S2.** Multivariable Cox regression analysis (Doubly robust estimation) for 28-day mortality in the propensity score-matched critically ill cohort (n=132).

| Variables in the Model | Unadjusted Model (Matched Cohort) | *p*-value | Adjusted Model (Doubly Robust) | *p*-value |
| --- | --- | --- | --- | --- |
|  | HR (95% CI) |  | HR (95% CI) |  |
| Ulinastatin treatment (Yes vs. No) | 0.51 (0.30 - 0.87) | 0.011 | 0.46 (0.26 - 0.82) | 0.009 |
| Timing to ICU admission (per 1-day increase) | - | - | 1.01 (0.97 - 1.04) | 0.695 |
| Vasopressor requirement (Yes vs. No) | - | - | 1.20 (0.67 - 2.12) | 0.540 |
| ARDS severity (Overall) | - | - | - | <0.001 |
| Moderate vs. Mild | - | - | 4.82 (1.11 - 20.95) | 0.036 |
| Severe vs. Mild | - | - | 15.40 (3.62 - 65.45) | <0.001 |
| Bacterial co-infection (Yes vs. No) | - | - | 0.93 (0.51 - 1.71) | 0.812 |
